# Platelet count and hypertension as indicators of height loss in the general population: A prospective study

**DOI:** 10.1371/journal.pone.0314527

**Published:** 2024-12-02

**Authors:** Yuji Shimizu, Hirotomo Yamanashi, Yuko Noguchi, Shin-Ya Kawashiri, Kazuhiko Arima, Yasuhiro Nagata, Takahiro Maeda

**Affiliations:** 1 Department of General Medicine, Nagasaki University Graduate School of Biomedical Sciences, Nagasaki, Japan; 2 Epidemiology Section, Division of Public Health, Osaka Institute of Public Health, Osaka, Japan; 3 Leading Medical Research Core Unit, Nagasaki University Graduate School of Biomedical Sciences, Nagasaki, Japan; 4 Department of Community Medicine, Nagasaki University Graduate School of Biomedical Sciences, Nagasaki, Japan; 5 Department of Public Health, Nagasaki University Graduate School of Biomedical Sciences, Nagasaki, Japan; University of Montenegro-Faculty of Medicine, MONTENEGRO

## Abstract

Circulating CD34-positive cell count is inversely associated with height loss. It acts as an indicator of endothelial repair activity. In conjunction with CD34-positive cells, platelets contribute to endothelial repair. The presence of hypertension increases the demand for endothelial repair. Therefore, platelet count could be associated with height loss among individuals with hypertension. A retrospective study of 2,343 individuals aged 40 to 79 years was conducted. Height loss was defined as being in the highest quartile of annual height decrease (1.6 mm/year for men and 2.0 mm/year for women). A significant inverse association between platelet count and height loss was observed only among participants with hypertension. After adjusting for known cardiovascular risk factors, the odds ratio (95% confidence interval) for height loss per 1 standard deviation increment in platelet count (5.09×10^4^/μL for men and 5.03×10^4^/μL for women) was 0.83 (0.70, 0.98) for participants with hypertension and 1.02 (0.90, 1.16) for participants without hypertension. Independent of known cardiovascular risk factors, platelets could prevent accelerated height loss among individuals with hypertension. Unlike CD34-positive cell count, platelet count and blood pressure, which are easy to assess in daily clinical practice, influence height loss.

## Introduction

Independent of known cardiovascular risk factors, circulating CD34-positive cell count is significantly inversely associated with height loss among Japanese individuals aged 60–69 years [1]. Since circulating CD34-positive cell count could act as an indicator of endothelial repair activity [2], higher endothelial repair activity might prevent height loss.

Height loss starting in middle age is an independent risk factor for cardiovascular mortality among the elderly [3] while circulating CD34-positive cell count is also reported to be inversely associated with cardiovascular mortality [4, 5]. Therefore, CD34-positive cells could be contributing to the prevention of height loss related to cardiovascular mortality. However, measuring circulating CD34-positive cells is not easy in daily clinical practice.

In conjunction with platelets, CD34-positive cells contribute to endothelial repair [6, 7]. Platelet count, which is easy to measure in daily clinical practice, could act as an indicator of endothelial repair activity [8]. Endothelial repair is required when the endothelium is injured. Hypertension, which strongly injures the endothelium, is a well-established cause of death from cardiovascular disease in the general Japanese population [9]. Therefore, hypertension status might influence the association between platelet count and height loss in the general population.

We hypothesized that platelet count is significantly inversely associated with height loss only among individuals with hypertension. To test our hypothesis, we conducted a prospective study of 2,343 Japanese individuals aged 40–79 years with mean follow-up of 3.4 years (standard deviation [SD], 1.2 years).

## Methods

### Study population

The study population comprised 2,977 residents of Goto city or rural communities in western Japan aged 40–79 years who underwent an annual medical check-up from 2014–2016, which was considered the baseline evaluation. Participants without platelet count data (n = 35), blood pressure data (n = 4), or data on smoking habits (n = 5) and drinking habits (n = 1) were excluded. We also excluded 589 individuals who did not undergo an annual health check-up during the follow-up period (2015–2019). The remaining participants, 2,343 individuals aged 67.9 ± 7.8 years (range, 40–79 years), were enrolled in the study.

All procedures performed in this study were in accordance with the ethical standards of the institutional research committee and the 1964 Declaration of Helsinki and its later amendments. Written consent forms were used to ensure that participants understood the objectives of the study when obtaining informed consent. The study was approved by the Ethics Committee of Nagasaki University Graduate School of Biomedical Sciences (project registration number: 14051404). Data for research purposes was accessed on 10 April 2024. No personally identifiable information was available to any of the authors.

### Data collection and laboratory measurements

Trained interviewers obtained information on clinical characteristics. Body weight and height were measured using an automatic body composition analyzer. Body mass index (BMI) was calculated.

Systolic and diastolic blood pressure were measured in the right arm after at least 5 minutes of rest, in a sitting position, with a blood pressure measuring device (HEM-907; Omron, Kyoto, Japan) and recorded by trained examiners. Hypertension was defined as systolic blood pressure ≥140 mmHg, diastolic blood pressure ≥90 mmHg, or both.

Fasting blood samples were collected in ethylenediaminetetraacetic acid—2 potassium (EDTA-2K) and siliconized tubes. Serum high-density lipoprotein cholesterol (HDLc), serum triglycerides (TG), and hemoglobin A1c (HbA1_C_) were measured using standard laboratory procedures at SRL, Inc. (Tokyo, Japan).

Carotid intima-media thickness (CIMT) was measured with ultrasonography of the left and right carotid arteries by experienced vascular technicians using a LOGIQ Book XP device with a 10-MHz transducer (GE Healthcare, Milwaukee, WI, USA). The maximum values for the left and right common carotid artery CIMT were calculated using digital edge-detection software (Intimascope; MediaCross, Tokyo, Japan) with a previously described protocol [10]. Intimascope is an innovative software developed for CIMT measurement to minimize measurement errors. This software makes it possible to semi-automatically recognize the edges of the internal and external membranes of the blood vessels. It semi-automatically determines the distance at a sub-pixel level (estimated to be 0.01 mm) using a polynomial measurement formula [11].

Height loss was defined as being in the highest quartile of height decrease per year (decrease of 1.6 mm/year for men and 2.0 mm/year for women), as in a previous study [1].

### Statistical analysis

The characteristics of the study population in terms of platelet count and hypertension status are presented as means ± SD or n (%), except for TG. Since TG values had a skewed distribution, logarithmic transformation was performed and the median (interquartile range) was presented.

Logistic regression models were used to calculate odds ratios (ORs) and 95% confidence intervals (CIs) to determine the hypertension-specific association between platelet count and height loss. The effect of hypertension on the association between platelet count and height loss was also evaluated.

Height loss is inversely associated with levels of circulating CD34-positive cells [1] that contribute to endothelial repair [2], including CIMT progression [12]. Therefore, three different models were used to calculate ORs and 95% CIs. Model 1 only adjusted for sex and age. Model 2 further adjusted made for CIMT. Finally, Model 3 further adjusted for other potential confounding factors, namely systolic blood pressure (mmHg), BMI (kg/m^2^), drinking status (non-drinker, often drinker, or daily drinker), smoking status (never smoker, former smoker, or current smoker), HDLc (mg/dL), TG (logarithmic value), and HbA1c (%).

For sensitivity analysis, the hypertension-specific association between platelet count and height loss was evaluated with another definition of height loss, i.e., being in the highest tertile of height decrease per year. Furthermore, sex-specific analysis of the main associations was also performed.

All statistical analyses were performed with SAS for Windows, version 9.4 (SAS Inc., Cary, NC). As in previous studies [13, 14], values of p < 0.05 for main effects and p < 0.2 for interactions were considered statistically significant.

## Results

### Characteristics of the study participants by platelet count

Table 1 shows the clinical characteristics of the study participants by platelet count. Platelet count was significantly inversely associated with age, and CIMT and significantly positively associated with diastolic blood pressure, current smoker, and TG levels.

**Table 1:** Characteristics of the study population by platelet count quartile.

	Platelet count (Quartiles)				*p*
	Q1	Q2	Q3	Q4	
	(Lowest)	(Lower)	(Higher)	(Highest)	
Number of participants	585	587	592	579	
Men, %	37.4	37.3	38.0	38.0	0.992
Height (men), cm (n)	163.7 ± 5.7 (219)	163.5 ± 6.6 (219)	163.8 ± 6.0 (225)	164.7 ± 6.9 (220)	0.611
Height (women), cm (n)	151.5 ± 5.8 (366)	151.6 ± 5.3 (368)	151.4 ± 5.7 (367)	151.2 ± 6.1 (359)	0.765
Age, years	69.6 ± 7.2	68.1 ± 7.5	67.7 ± 7.5	66.0 ± 8.6	<0.001
CIMT, mm	0.94 ± 0.23	0.95 ± 0.24	0.93 ± 0.21	0.91 ± 0.22	0.022
BMI, kg/m^2^	23.0 ± 3.6	23.3 ± 3.2	23.3 ± 3.2	23.3 ± 3.2	0.188
SBP, mmHg	132 ± 17	132 ± 16	133 ± 17	133 ± 16	0.324
DBP, mmHg	76 ± 11	77 ± 11	78 ± 11	78 ± 11	0.029
Daily drinker, %	15.6	15.0	20.4	15.5	0.040
Often drinker, %	10.9	13.5	9.5	13.8	0.064
Current smoker, %	7.0	7.7	10.6	13.3	<0.001
Former smoker, %	22.1	20.8	24.3	22.8	0.529
TG, mg/dL	84	90	95	98	<0.001*^2^
	[60, 117]*^1^	[67, 121]*^1^	[68, 133]*^1^	[70, 142]*^1^	
HDLc, mg/dL	61 ± 15	60 ± 15	61 ± 15	60 ± 14	0.231
HbA1c, %	5.70 ± 0.56	5.69 ± 0.48	5.71 ± 0.52	5.75 ± 0.57	0.221

Values are means ± standard deviation unless otherwise indicated.

*1: Values are medians [interquartile quartile].

*2: Logarithmic transformation was performed. CIMT: carotid intima-media thickness. BMI: body mass index. SBP: systolic blood pressure. DBP: diastolic blood pressure. TG: triglycerides. HDLc: high-density lipoprotein cholesterol. HbA1c: glycated hemoglobin. Quartiles of platelets levels for men were <18.4 ×10^4^/μL for Q1 (Lowest), 18.4–21.3 ×10^4^/μL for Q2 (Lower), 21.4–24.7×10^4^/μL for Q3 (Higher), and ≥24.8×10^4^/μL for Q4 (Highest). For women, the corresponding values were <19.5×10^4^/μL for Q1 (Lowest), 19.6–22.6×10^4^/μL for Q2 (Lower), 22.7–26.2×10^4^/μL for Q3 (Higher), and ≥26.3×10^4^/μL for Q4 (Highest).

### Characteristics of the study participants by hypertension status

The clinical characteristics of the study population by hypertension status are shown in Table 2. Compared to participants without hypertension, a higher proportion of participants with hypertension were men, daily drinkers, and former smokers, respectively. Participants with hypertension had higher age, CIMT, BMI, SBP, DBP, and TG levels than those without hypertension.

**Table 2:** Characteristics of the study participants by hypertension status.

	Hypertension		*p*
	(-)	(+)	
Number of participants	1552	791	
Men, %	35.9	41.2	0.012
Age, years	67.3 ± 8.1	69.0 ± 7.2	<0.001
Height (men), cm (n)	164.0 ± 6.4 (557)	163.6 ± 6.2 (326)	0.387
Height (women), cm (n)	151.5 ± 5.7 (995)	151.2 ± 5.6 (465)	0.182
CIMT, mm	0.92 ± 0.22	0.97 ± 0.22	<0.001
BMI, kg/m^2^	22.9 ± 3.2	23.9 ± 3.4	<0.001
SBP, mmHg	123 ± 10	150 ± 12	<0.001
DBP, mmHg	73 ± 8	86 ± 11	<0.001
Daily drinker, %	14.6	20.6	<0.001
Often drinker, %	12.5	10.7	0.215
Current smoker, %	9.9	9.2	0.026
Former smoker, %	20.7	26.0	0.003
TG, mg/dL	88 [64, 122]*^1^	99 [71, 135]*^1^	<0.001*^2^
HDLc, mg/dL	61 ± 15	60 ± 15	0.788
HbA1c, %	5.70 ± 0.52	5.74 ± 0.56	0.221

Values are means ± standard deviation unless otherwise indicated.

*1: Values are medians [interquartile quartile].

*2: Logarithmic transformation was performed. CIMT: carotid intima-media thickness. BMI: body mass index. SBP: systolic blood pressure. DBP: diastolic blood pressure. TG: triglycerides. HDLc: high-density lipoprotein cholesterol. HbA1c: glycated hemoglobin.

### Association between platelet count and height loss by hypertension status

Independent of known cardiovascular risk factors, platelet count was significantly inversely associated with height loss among participants with hypertension but not among those without hypertension (Table 3). The fully adjusted OR (95% CI) for height loss with each 1 SD increment in platelet count was 0.83 (0.70, 0.98) for participants with hypertension and 1.02 (0.90, 1.16) for participants without hypertension.

**Table 3:** Association between platelet count and height loss by hypertension status.

		Platelet count (Quartiles)				p	1 SD increment in platelet count
		Q1 (Lowest)	Q2 (Lower)	Q3 (Higher)	Q4 (Highest)		
Hypertension (-)							
	Number of participants	393	407	383	369		
	Number of height loss (%)	102 (26.0)	93 (22.9)	95 (24.8)	81 (22.0)		
	Model 1	Ref	0.90 (0.65, 1.25)	1.03 (0.74, 1.43)	0.94 (0.67, 1.32)	0.921	1.01 (0.89, 1.14)
	Model 2	Ref	0.90 (0.65, 1.25)	1.03 (0.74, 1.43)	0.94 (0.67, 1.32)	0.914	1.01 (0.89, 1.14)
	Model 3	Ref	0.89 (0.64, 1.24)	1.05 (0.75, 1.46)	0.94 (0.67, 1.34)	0.999	1.02 (0.90, 1.16)
Hypertension (+)							
	Number of participants	192	180	209	210		
	Number of height loss (%)	68 (35.4)	49 (27.2)	50 (23.9)	48 (22.9)		
	Model 1	Ref	0.69 (0.44, 1.08)	0.60 (0.39, 0.93)	0.59 (0.38, 0.92)	0.015	0.83 (0.71, 0.98)
	Model 2	Ref	0.67 (0.43, 1.05)	0.60 (0.39, 0.93)	0.59 (0.38, 0.92)	0.016	0.83 (0.71, 0.98)
	Model 3	Ref	0.70 (0.45, 1.10)	0.59 (0.38, 0.92)	0.59 (0.38, 0.93)	0.015	0.83 (0.70, 0.98)

Model 1 adjusted for sex and age. Model 2 adjusted for sex, age, and carotid intima-media thickness (CIMT). Model 3 adjusted for sex, age, body mass index, drinking status, smoking status, systolic blood pressure (SBP), triglycerides (TG), high-density lipoprotein cholesterol (HDLc), and glycated hemoglobin (HbA1c). Height loss was defined as being in the highest quartile of height decrease per year. Ref: Reference; SD, standard deviation. Quartiles of platelets levels for men were <18.4 ×10^4^/μL for Q1 (Lowest), 18.4–21.3 ×10^4^/μL for Q2 (Lower), 21.4–24.7×10^4^/μL for Q3 (Higher), and ≥24.8×10^4^/μL for Q4 (Highest). For women, the corresponding values were <19.5×10^4^/μL for Q1 (Lowest), 19.6–22.6×10^4^/μL for Q2 (Lower), 22.7–26.2×10^4^/μL for Q3 (Higher), and ≥26.3×10^4^/μL for Q4 (Highest). The 1 SD increment in platelet count was 5.09×10^4^/μL for men and 5.03×10^4^/μL for women.

### Influence of hypertension on the association between platelet count and height loss

Hypertension had a significant effect on the association between platelet count and height loss. The adjusted p value was 0.062 for Model 1, 0.061 for Model 2, and 0.064 for Model 3.

### Sex-specific findings

Essentially the same associations were observed for men and women. Among participants with hypertension, the OR (95% CI) in the model that adjusted for known cardiovascular risk factors (Model 3) for height loss per 1 SD increment in platelet count was 0.80 (0.61, 1.04) for men (n = 326) and 0.84 (0.68, 1.05) for women (n = 465). Among participants without hypertension, the corresponding values were 1.03 (0.84, 1.28) for men (n = 557) and 1.02 (0.87, 1.19) for women (n = 995).

### Hypertension-specific association between height loss defined as being in the highest tertile of height decrease per year and platelet count

For sensitivity analysis, the hypertension-specific association between height loss defined as being in the highest tertile of height decrease per year and platelet count was also calculated; the result was essentially the same. In the fully adjusted model (Model 3), the OR (95% CI) for height loss with each 1 SD increment in platelet count was 0.82 (0.70, 0.96) for those with hypertension and 1.11 (0.99, 1.25) for those without hypertension.

## Discussion

The major finding of the present study is that independent of known cardiovascular risk factors, platelet count is inversely associated with height loss among individuals with hypertension but not among those without hypertension.

However, the mechanism underlying those associations has not been clarified yet. Sex might not affect the hypertension-specific association between platelet count and height loss because the sensitivity analysis found essentially the same associations in both sexes.

A previous study of men aged 60 to 69 years showed a significant inverse association between height loss defined as being in the highest quartile of height decrease per year and circulating CD34-positive cell count [1]. After adjusting for known cardiovascular risk factors, the OR (95% CI) for height loss with circulating CD34-positive cell count (logarithmic values) was 0.49 (0.32, 0.74). Inappropriate endothelial repair related to a shortage of circulating CD34-positive cells [2, 15] might be a significant risk factor for height loss. In other words, appropriate endothelial repair related to having a sufficient circulating CD34-positive cell count could prevent height loss.

In conjunction with hematopoietic stem cells known as CD34-positive cells, platelets play an important role in endothelial repair [6, 7], including the development of atherosclerosis [6, 16]. Thus, platelet count could act as an indicator of endothelial activity [8] and higher levels of endothelial repair activity might have the beneficial effect of preventing height loss. However, in the present study, an inverse association between platelet count and height loss was observed only among participants with hypertension.

Hypertension is strongly associated with endothelial repair activity [2, 17]. In this study, participants with hypertension had more cardiovascular risk factors than participants without hypertension. Therefore, to maintain vascular health among individuals with hypertension, active endothelial repair is necessary. Since platelet count could indicate endothelial repair activity [8], platelet count could be inversely associated with height loss among individuals with hypertension.

However, mechanisms that explain the association between inappropriate endothelial repair and height loss in the general population have not yet been clarified. The main reasons for height loss among adults are intervertebral disc degeneration and vertebral fracture. Disturbance of blood flow to intervertebral discs is an important part of the pathogenesis of intervertebral disc degeneration [18]. Disruption of the microcirculation is associated with many bone diseases, including osteoporosis [19]. Therefore, maintenance of the microcirculation could be important to preventing intervertebral disc degeneration and osteoporosis. Since CD34-positive cells also play an important role in the development of angiogenesis [20], they contribute to maintenance of the microcirculation [2, 21]. In conjunction with circulating CD34-positive cells, platelets contribute to endothelial repair [6, 7]; higher platelet counts in individuals with hypertension might indicate the presence of appropriate maintenance of the microcirculation related to the prevention of intervertebral disc degeneration and vertebral fracture.

Although circulating CD34-positive cell count is inversely associated with height loss [1], measuring CD34-positive cells is not easy in daily clinical practice. In the present study, platelets, which contribute to endothelial repair in conjunction with CD34-positive cells, were inversely associated with height loss among participants with hypertension. Since blood pressure and platelet count are easy to measure, they might be efficient ways to estimate the risk of height loss in daily clinical practice. In addition, because the present study focused on a biological marker related to active endothelial repair, our present findings can help clarify novel mechanisms that induce height loss.

Limitations of the present study warrant consideration. Because most cases of intervertebral disc degeneration and vertebral compression fracture, which are the main causes of height loss, are asymptomatic [22, 23], we could not evaluate the causes of height loss using a questionnaire. To evaluate causes of height loss, imaging data from plain radiography, computed tomography, or magnetic resonance imaging are necessary. While being in the highest quartile of height decrease per year was defined as height loss in the present study, an efficient cutoff point for defining height loss has not been established. However, additional analysis with height loss defined as being in the highest tertile of height decrease per year showed essentially the same associations.

Height loss starting in middle age is associated with mortality from cardiovascular disease [3]. The present study indicates that insufficient endothelial repair might be an independent risk factor for height loss. Since circulating CD34-positive cell count is inversely associated with mortality from cardiovascular disease [4, 5], the present study suggests that height loss and hypertension status could be efficient ways to evaluate endothelial repair activity. Further investigation of this concept is necessary.

## Conclusion

Independent of known cardiovascular risk factors, platelet count is inversely associated with height loss only in individuals with hypertension. Although further investigation is necessary, the present findings indicate that height loss and the presence of hypertension might act as indicators of endothelial repair activity.
